# National Diagnostic Reference Level (DRL) for nuclear medicine computed tomography–positron emission tomography hybrid imaging studies for kuwait population: second phase dose audit-2019

**DOI:** 10.1259/bjro.20210020

**Published:** 2021-09-19

**Authors:** Michael Masoomi, Iman Al-Shammeri, Latifah Al-Kandari, Hany Elrahman, Jehan Al-Shammeri

**Affiliations:** ^1^ Nuclear Medicine and Molecular Imaging Section, Department of Medical Imaging, Adan Hospital, MOH, Hadiya, Kuwait; ^2^ Radiology Section, Department of Medical Imaging, Adan Hospital, MOH, Hadiya, Kuwait; ^3^ Nuclear Medicine Department, Faculty of Medicine, Kuwait University, Kuwait City, Kuwait

## Abstract

**Objective:**

Diagnostic reference levels (DRLs) for CT part of positron emission tomography-CT (PET-CT) examinations are limited. The study was aiming to execute the second phase of the national DRL in support of optimisation and dose reduction in State of KW.

**Methods:**

In this multicentre collaborative PET-CT study, oncology patient data were exclusively collected due to the National MOH Ethical Committee recommendation and limitation of the other studies. Median, Mean, SD, 75th, 25th percentiles as well as whole body (WB) effective dose (ED) were calculated. The study was UK-IPEM-based methodology and it was the second phase of the study in Kuwait.

**Results:**

Half body (HB) and WB scans were 65 and 35% of the total enterers (309). The third quartile dose–length product (DLP) (mGy x cm) and volumetric CT dose index (mGy) values for the HB (537, 5) were higher than the UK NDRL (400, 4.3) but were lower than the Swiss NDRL (620, 6) and the France NDRL (762, 7.7). Comparatively, the proposed NDRLs for the WB (684, 4.1) were lower than Swiss National Data (720, 5.0) though, the Swiss had about 5000 (HB) & 706 (WB), the UK had 370 (HB) and France had 1000 (HB) entries. Calculated ED varied from 4.1 to 10.2 mSv, (mean values = 6.9 mSv) for HB and from 2.6 to 7 mSv (mean value = 4.6 mSv).

**Conclusions:**

There was 9.1% improvement in NDRL for 2019, compared to 2018, but there is a continuous need for improving NDRL.

**Advances in knowledge:**

Data provided a trend of NDRL that is served as a national data bank for continuous optimisation.

## Introduction

There is an increased global focus on the need to carefully manage radiation exposures from CT imaging.^1^ Recent development of positron emission tomography-CT (PET-CT) systems has allowed concurrent or with minimum time delay to perform anatomic and functional imaging of organs.^2–4^ The CT components of PET-CT systems are equivalent in power output to their standalone versions and may be used for diagnostic purposes under appropriate CT technique. In addition to diagnostics acquisition, the application of CT in hybrid PET-CT scanners may serve other speciﬁc purposes, including attenuation correction of the PET image data and tissue localisation.^5^ The CT dose measurement concept is based on the CT dose index (CTDI), which represents the average absorbed dose of irradiation of contiguous slices.

On contrary to diagnostic CT, published dose reference levels for CT used in hybrid PET-CT examinations and guidance on CT dosimetry metrics in the literature on nuclear medicine practice standards are limited, and many of available reports reference to dedicated diagnostic CT practice standards, which may not be appropriate for CT in PET-CT. Of the limited documentation on CT technique for PET oncology, there is a general acceptance that CT dose is tailored to its purpose in the reconstruction or the interpretation process.^6,7^ Furthermore, a widely accepted approach to optimisation of medical radiation exposures, recommended by the ICRP^8,9^ and the International Atomic Energy Agency (IAEA),^10^ is the establishment and use of national, regional and local diagnostic reference levels (DRLs). DRLs of the volumetric CT dose index (CTDI_vol_) which are levels for whole body/half body (WB/HB) CT used in PET-CT examinations are limited.^2^ Examination or procedure-specific DRLs can provide the stimulus for monitoring practice to promote improvements in patient protection.^11^

DRLs should be set for representative examinations or procedures performed in the local area, country or region where they are applied. National DRLs (NDRLs) should be set on the basis of wide scale surveys of the median doses representing typical practice for a patient group (*e.g.* adults or children of different sizes) at a range of representative healthcare facilities for a specific type of examination or procedure. NDRLs are commonly set at the third quartile values (the values that splits off the highest 25% of data from the remaining 75%) of these national distributions.^12^ As such, NDRLs are not optimum doses, but nevertheless they are helpful in identifying potentially unusual practice, where median doses are among the highest 25% of the national dose distribution. DRLs can be also established for a region within the country or, in some cases, regions of several countries. Clinical protocols for performing a particular examination or procedure should be reviewed if the comparison shows that the facility’s typical dose exceeds the DRL, or that the facility’s typical dose is substantially below the DRL and it is evident that the exposures are not producing images of diagnostic usefulness or are not yielding the expected medical benefit to the patient.

There is no preferred custodian: what is important is that a patient dose database (for DRLs) is established and maintained, DRL values are set, disseminated through the regulatory processes, and a process for periodic review is established.

In this study, DRLs for each centre (no:8) having PET-CT were calculated based on the local practice and then an NDRL for oncology examinations (majority of studies) and patients group were proposed adopting the third quartile value of the CTDIvol and dose–length product (DLP) following the wide scale national surveys according to the IPEM-UK frame work and the ICRP recommendation for the state of Kuwait. A comparative study to this project were a national survey conducted by the UK (47 PET-CT centres), Swiss (16 PET-CT centres) and France (56 PET-CT centres) on PET-CT oncologic procedures. The study was reviewed and approved by the medical ethics committee of MOH, Kuwait and all study participants provided informed written consent about personal and medical data collection prior to study enrollment.^13–15^

## Methods and materials

In this second phase continuing multicentre collaborative study, a multiple CT data of PET-CT hybrid imaging system that were in practice in Kuwait Hospitals were collected and analysed to set up a NDRL base line (Supplementary Material 1). The data collection was restricted to adult oncology patients as per the Kuwait Ethical Committee recommendation and the limitation of the other studies. The Methodology, based on the UK–IPEM, was adopted as per the first-year audit to suit the proposed study involving the KW NM Clinical Centres. The UK methodology was initiated in support of optimisation and data were acquired prospectively or retrospectively. A ‘protocol guidance’ page provided details of typical examinations to search for and a help-sheet was available for different scanners which showed you where to find the value parameters on the scanners. For each scanner, a minimum of 10 different patient data (but ideally 20–30 patients) were collected.^13^

The studies were carried out with participation of 8 PET-CT centres and 309 oncology patient cases in comparison to 197 patients in the first year (2018) where 7 centres were participated. The focus was on all PET-CT imaging systems and procedures, regardless of their locations and numbers of the systems availability in one centre. There was a variation for the proposed and achievable local DRL in practice between seven centres, highlighting the need for continued assessment of national DRL and to monitor the trend of the variation with an intention to minimise the radiation dose and its impact on Kuwaiti patients.^1^

To minimise the influence from centres that provided a significantly large number of entries compared to others, the limiting data contribution from each centre was set to a maximum of 40 entries. In addition, all topograms (scanograms) and monitoring steps used in contrast-enhanced CT acquisitions (if any) were excluded from the analysis. The priority was to estimate typical patient dose quantities for the common present practice on adult patients according to the following steps:

- To record displayed values of radiation dose quantity for maximum samples of 40 typical adult patients per centre (*i.e.* 309 sample data for all the centres), undergoing procedures for oncology clinical indications. For CT part, the clinical purpose of exposure, attenuation correction (AC), localisation or diagnosis for the PET-CT imaging centres were recorded for WB and HB.
- To calculate, for each type of examination, the median values of dose quantities (*e.g.* CTDI_vol_ and DLP for CT); which were the typical dose levels (but were not the local DRLs that are set for a group of imaging systems or a group of hospitals).
- To compare the typical dose levels (median values) with the published DRLs for a similar practice in the absence of local or national DRLs (UK, Swiss and France), in order to provide a broad indication of our relative performance and urgency of need for improvement in our imaging technique. A proposed national DRL for Kuwait was then suggested.
- The effective dose for the oncology examination at each centre, using CT part of PET-CT was calculated based on the measured dose values.

### Statistical analysis

Statistical analysis was performed on the data collected for the oncology protocol from the participating centres, in respect of CTDI*vol*, DLP and scan length (SL), taking into consideration the intended aim (attenuation correction and localisation) declared by each nuclear medicine centre. For the each of these metrics, the number of entries, median, mean, standard deviation, minimum and maximum values and 75th & 25th percentiles of the combined were calculated. Rounded third quartile and first quartile values of CTDIvol and DLP were used to produce suggested NDRLs, and to produce achievable doses respectively as a further aid to optimisation.^16^

NDRLs representing the 75th% percentile of the data distribution, were proposed in addition to achievable dose (defined as the 25th% percentile of the data distribution) for each protocol. The ICRP suggests taking the third quartile of the distribution of individual median values as the DRL. However, in this study, we present both mean and median for DRL to accommodate suggestions by the various groups, including the UK, Swiss and France national surveys. All the eight centres in KW, except one, used automatic exposure control (AEC) that modulates radiation exposure automatically and is widely used for optimisation of radiation dose in CT.^17–19^

We also estimated the effective dose (ED) as a pre-requisite for optimisation and monitoring of radiation exposure of the CT part of PET/CT facilities. ED is often estimated as a product of the DLP value and a conversion factor selected according to the imaging region.^20–22^ CTDIvol is calculated on the basis of radiation dose measured in imaging 16 cm and 32 cm CT dosimetry phantoms for head-mode and body-mode imaging, respectively. The conversion factor from DLP to ED depends on the location, size, and radiosensitivity of organs and tissues exposed to radiation and is lower for the head than for the trunk. For ^18^F-FDG PET-CT oncology applications, CT images are usually acquired from the head to the proximal thigh sequentially, and a single DLP value, representing half body radiation exposure, is provided on a scanner.

It is also important to note, whereas the fundamental concept of ED has not changed with new ICRP recommendations, important aspects of its calculation have been updated, leading in particular to changes in values of dose per unit exposure since the previous UK CT survey for 2003.

To assess the radiation dose from the CT component of the examination, we used DLP values from the scanner-generated dose reports and a conversion factor—*i.e.* the region-specific normalised effective dose per DLP (mSv ×mGy^−1^ × cm^−1^) conversion factor (*k*): *ED* (mSv) ≈ *k* × *DLP*. For the HB and WB scan, we used a **k** value of 0.015 mSv ×mGy^−1^ × cm^−1^ and 0.0093 mSv ×mGy^−1^ × cm^−1^ respectively.^23,24^ The coefficients of ED/DLP for examinations were for adult patients and were calculated as mean values, over a range of CT scanner models operating at medium applied potentials (principally 120 kVp), on the basis of ICRP 103 tissue weighting factors and ICRP 110 voxel phantoms (as an average for AM and AF).^25^

For the calculation of displaying CTDIvol and DLP; GE scanners (GE, Milwaukee, WI) is using 32 cm body CTDI phantom on all the systems in this study. Patients were not categorised by age, sex, and weight as the AEC for all the scans, which accounted for differences in patient size. All PET-CT centres in this study, except one, used the Adaptive Statistical Iterative Reconstruction (ASiR) that has potential to achieve significant reductions in patient radiation dose in CT exams while achieving image reconstruction speed similar to that of filtered back projection (FBP).

### International comparison

The “mean and median” values of dose quantities (CTDI_vol_ and DLP for CT) for data, collected from the Kuwait multiple centres to establish whether they are above or below the published DRL. The “mean” values had been recommended earlier, but the recent recommendations are favouring median values.^26–28^ A similar work by the CT working groups in the UK, Swiss and France have recently been performed and the results have been published.^13–15^ Some of the protocols exercised in Kuwait are common and thus DRL’s can be directly compared.

## Results

The HB (chest, abdomen and pelvis) fluorine-18-fludeoxyglucose (^18^F-FDG or ^F^18-NAF) oncology scanning comprised the majority of PET-CT imaging procedures (65% of total collected data for 2019 *vs* 53% for the earlier 2018 audit), though there was much variations in half body studies in the centres. The whole body PET-CT examinations (head to toe) were 35% in 2019 in contrast to 47% in 2018. Maximum variation for the “mean” SL value (cm) between eight PET-CT centres was 9% for HB and 11% for WB PET/CT examinations (Supplementary Materials 2 and 3).

Summary of dose and scan length statistics for the combined scan length (HB + WB) were presented in Table 1.

**Table 1.** Summary statistics for the distribution of the scanner volume CT dose index and dose–length product for the protocol list (WB + HB) of each centre using PET-CT

Centre	Protocol application (AC&L)	CTDI_vol_ (mGy)					DLP (mGy × cm)					Scan length (cm)				
		Median	Mean	STD	Min	Max	Median	Mean	STD	Min	Max	Median	Mean	STD	Min	Max
1	PET Oncology [WB + HB]: 40N	3.6	3.6	1.1	1.6	7.9	453	448	103	125	908	125	130	34	74	180
2	PET Oncology [WB + HB]:31N	4.4	4.4	1.2	2.3	8.4	513	510	122	101	969	109	118	24	98	180
3	PET Oncology [WB + HB]:40N	4.7	5.0	2.1	1.6	8.7	506	596	277	163	1501	109	122	31	97	180
4	PET Oncology [WB + HB]:40N	4.8	5.3	1.9	1.3	9.0	711	764	299	227	1657	156	141	28	97	180
5	PET Oncology [WB + HB]:40N	4.1	4.6	1.5	2.4	8.4	493	568	191	374	1108	109	123	33	97	180
6	PET Oncology [WB + HB]:38N	2.9	2.6	0.5	1.8	3.1	339	353	101	170	558	115	131	31	66	186
7	PET Oncology [WB + HB]:40N	2.7	2.7	0.9	1.4	5.0	277	306	110	144	666	99	113	26	86	185
8	PET Oncology [WB + HB]:40N	3.4	3.8	1.3	2.0	7.0	490	496	157	242	957	161	144	36	102	185

AC, Attenuation correction; CTDIvol, CT dose volume; DLP, Dose–length product; HB, Half body; L, Localisation; N, Number of entry; TB, Total body;WB, whole body.

There was a maximum of twofold variation in LDRL for CTDIvol and DLP between seven centres (Table 2). The proposed NDRL and achievable DRL (based on the median and mean values of CTDIvol and DLP) for HB, WB and HB + WB oncology examinations were calculated and presented in Table 3. Third quartile DLP (mGy x cm) and CTDIvol (mGy) values (537, 5) related to the Kuwait HB PET-CT scans (for setting NDRL) were higher than the current UK NDRL (400, 4.3) but lower than the Swiss National NDRL (620, 6) and the France National NDRL (762, 7.7). Comparatively, the proposed NDRLs for WB was (684, 4.1) which was lower than Swiss National Data (720, 5.0). The Kuwait results having a 200 (HB) and 109 (WB) entries were in agreement with the Swiss (5000 HB & 706 WB), the UK (370 HB) and France (1000 HB) entries (Table 4). The calculated ED varied from 4.1 to 10.2 mSv (mean value = 6.9 mSv) for HB and from 2.6 to 7 mSv (mean value = 4.6 mSv) for WB scans (Table 5).

**Table 2.** Proposed and achievable LDRL for AC and localisation product for the clinical NM examination protocol (WB + HB) at each centre using PET-CT

Centre	Protocol Application (AC&L)	Proposed LDRL (75th%)		Achievable DRL (25th%)	
		CTDIvol (mGy)	DLP (mGy × cm)	CTDIvol (mGy)	DLP (mGy × cm)
1	PET Oncology [WB + HB]:40N	4.2	520	2.9	382
2	PET Oncology [WB + HB]:31N	4.8	556	4.1	471
3	PET Oncology [WB + HB]:40N	7.0	792	2.9	406
4	PET Oncology [WB + HB]:40N	6.9	886	4.2	601
5	PET Oncology [WB + HB]:40N	5.0	607	3.8	448
6	PET Oncology [WB + HB]:38N	2.9	417	2.4	280
7	PET Oncology [WB + HB]:40N	3.1	366	2.1	223
8	PET Oncology [WB + HB]:40N	4.4	571	3.0	389

AC, Attenuation correction; CTDIvol, CT dose volume; DLP, Dose reference level; DLP, Dose length product; HB, Half body; L, Localisation; LDRL, Local dose reference level; TB, Total body;WB, whole body.

**Table 3.** Proposed and achievable NDRL for AC and localisation product for the suggested clinical NM protocols using PET-CT: (Based on Mean & Median Value)

Centre	Protocol application (AC&L)	Proposed LDRL (75th%)				Achievable DRL (25th%)			
		CTDIvol (mGy)		DLP (mGy × cm)		CTDIvol (mGy)		DLP (mGy × cm)	
1	PET Oncology [HB]:200N	5.0M	4.8M*	537M	514M*	3.8M	3.7M*	398M	409M*
2	PET Oncology [WB]:109N	4.1M	3.6M*	684M	536M*	2.9M	2.7M*	444M	444M*
3	PET Oncology [WB + HB]:309N	4.7M	4.5M*	575M	507M*	3.4M	3.3M*	424M	424M*

AC, Attenuation correction; CTDIvol, CT dose volume; DLP, Dose reference level; DLP, Dose length product; HB, Half body; L, Localisation; NDRL, National diagnostic reference level;WB, whole body.

**Table 4.** Comparison of proposed NDRL for AC and localisation product for the suggested clinical NM protocols using PET-CT: (Based on Mean & Median Values)

Centre	Protocol application (AC&L)	UK: Proposed NDRL		SWISS: Proposed NDRL		France: Proposed NDRL		KW: Proposed NDRL	
		CTDIvol (mGy)	DLP (mGy × cm]	CTDIvol (mGy)	DLP (mGy × cm)	CTDIvol (mGy)	DLP (mGy × cm)	CTDIvol (mGy)	DLP (mGy × cm)
1	PET Oncology [HB]:200N	4.3 M 4.3 M*	400 M 400 M*	6.0M 6.0M*	620 M 620 M*	6.6M 6.6M*	628 M 628 M*	5.0M 4.8M*	537 M 514 M*
2	PET Oncology [WB]:109N			5.0M 5.0M*	720 M 720 M*	7.7M 7.7M*	762 M 762 M*	4.1M 3.6M*	684 M 536 M*
3	PET Oncology [WB + HB]:309N							4.7M 4.5M*	575 M 507 M*

AC, Attenuation correction; CTDvol, CT dose volume; DLP, Dose–length product; DLP, Dose reference level; HB, Half body; L, Localisation; M, Mean; M*, Median; NDRL, National diagnostic reference level;WB, whole body.

**Table 5.** Comparison of CT effective dose as a result of AC and localisation product for the suggested clinical NM protocol using PET-CT: using the recommended published conversion factors; *K* = 0.015 (mSv/mGy/cm) & *K* = 0.0093 (mSv/mGy/cm)

	ED (mSv) Scan length - HB K-0.015 mSv/mGy/cm			ED (mSv) Scan length - WB K-0.0093 mSv/mGy/cm			ED (mSv) [HB +WB] K= [0.015 + 0.0093] mSv/mGy/cm		
		Mean	Median		Mean	Median		Mean	Median
1	PET Oncology [HB] : 25N	6.7	6.5	PET Oncology [WB] : 15N	4.3	4.4	PET Oncology [WB + HB]: 40N	5.5	5.5
2	PET Oncology [HB]: 25N	7.7	7.9	PET Oncology [WB]: 6N	4.0	4.1	PET Oncology [WB + HB]:31N	5.9	6.0
3	PET Oncology [HB]: 29N	7.7	8.4	PET Oncology [WB]: 11N	4.4	6.4	PET Oncology [WB + HB]:40N	6.1	7.4
4	PET Oncology [HB]: 18N	10.2	10.5	PET Oncology [WB]: 22N	7.0	7.6	PET Oncology [WB + HB]:40N	8.6	9.1
5	PET Oncology [HB]: 28N	7.2	7.8	PET Oncology [WB]: 12N	5.6	6.2	PET Oncology [WB + HB]:40N	6.4	7.0
6	PET Oncology [HB]: 24N	4.7	4.5	PET Oncology [WB]: 14N	4.3	4.1	PET Oncology [WB + HB]:38N	4.5	4.3
7	PET Oncology [HB]: 32N	4.1	4.3	PET Oncology [WB]: 8N	2.6	3.1	PET Oncology [WB + HB]:40N	3.4	3.7
8	PET Oncology [HB]: 19N	6.6	6.8	PET Oncology [WB]: 21N	4.8	4.9	PET Oncology [WB + HB]:40N	5.7	5.9

AC, Attenuation correction; CTDIvol, CT dose volume; DLP, Dose reference level; DLP, Dose–length product; HB, Half body; *K*, Conversion factor; L, Localisation; N, Number of entry; NDRL, National diagnostic reference level;WB, whole body.

**Figure 1.**
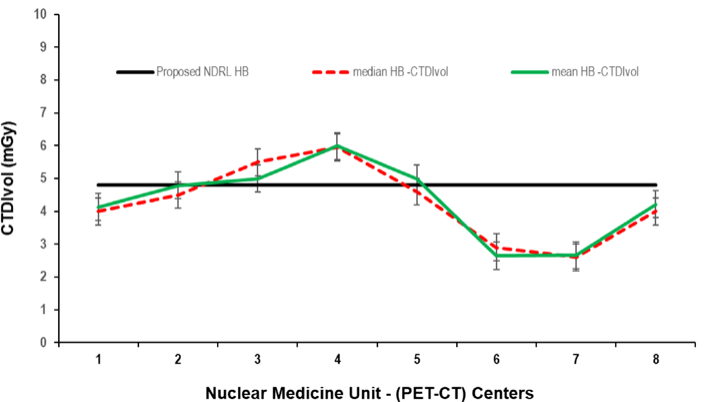
CTDIvol (75 th percentile) for each PET-CT unit, compared to the proposed NDRL for HB data. CTDI_vol_, volumetric CT dose index; PET, positron emission tomography; NDRL, national diagnostic reference level; WB, whole body.

**Figure 2.**
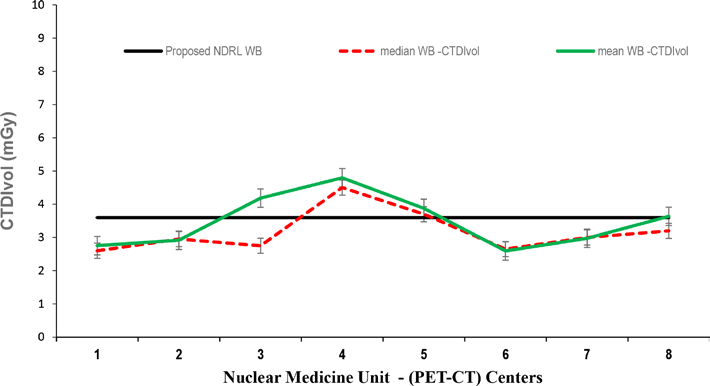
CTDIvol (75th percentile) for each PET-CT unit, compared to the proposed NDRL for WB data. CTDI_vol_, volumetric CT dose index; HB, half body; PET, positron emission tomography; NDRL, national diagnostic reference level; WB, whole body.

**Figure 3.**
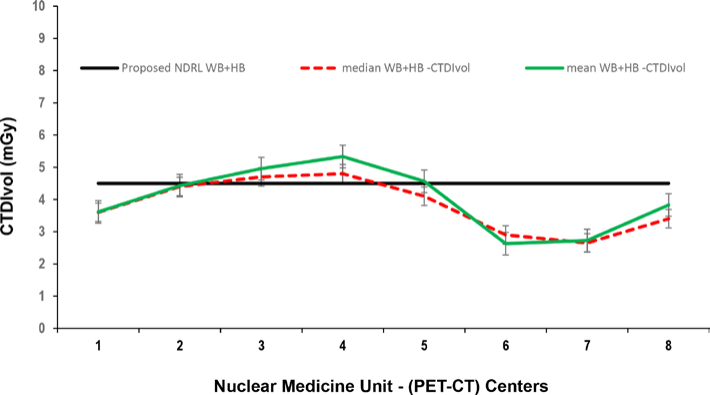
CTDIvol (75 th percentile) for each PET-CT unit, compared to the proposed NDRL for WB + HB data. CTDI_vol_, volumetric CT dose index; HB, half body; PET, positron emission tomography; NDRL, national diagnostic reference level; WB, whole body

**Figure 4.**
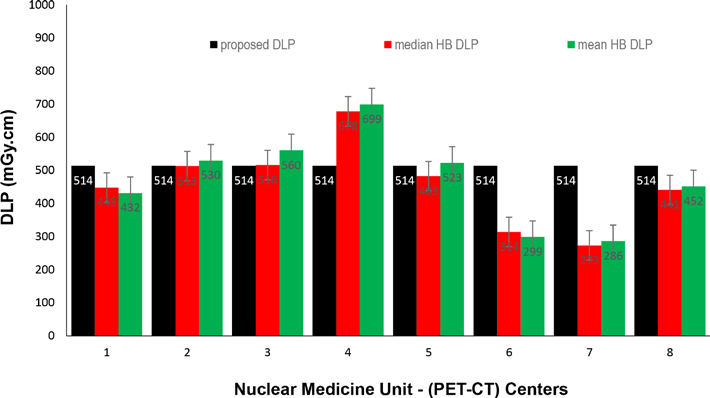
DLP (75th percentile) for each PET-CT unit, compared to the proposed NDLP for HB data. DLP, dose–length product; HB, half body; PET, positron emission tomography; NDRL, national diagnostic reference level.

**Figure 5.**
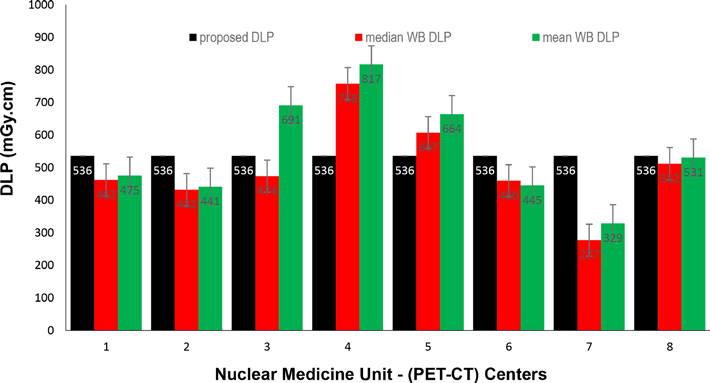
DLP (75th percentile) for each PET-CT unit, compared to the proposed NDRL for WB data. DLP, dose–length product; PET, positron emission tomography; NDRL, national diagnostic reference level; WB, whole body.

**Figure 6.**
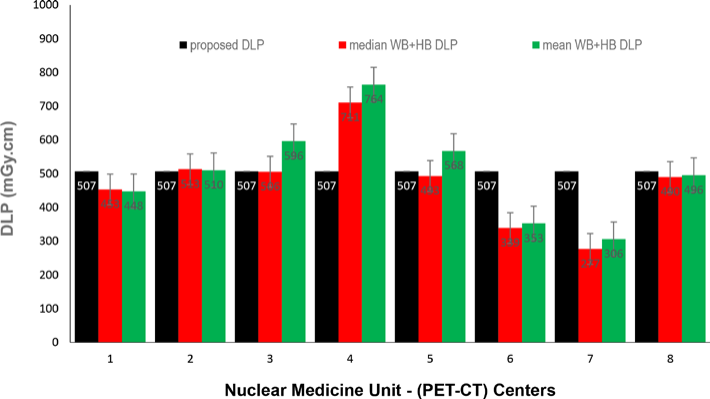
DLP (75th percentile) for each PET-CT unit, compared to the proposed NDRL for WB +HB data. DLP, dose–length product; HB, half body; PET, positron emission tomography; NDRL, national diagnostic reference level; WB, whole body.

Data presented in Figures 1–3 show the range of doses (75th percentile CTDIvol) for the proposed HB, WB and HB + WB oncology examinations related to AC & localisation clinical purposes. Similarly, Figures 4–6 present DLP variations (75th percentile) for HB, WB and WB + HB for each NM centre in relation to the proposed DLP. The dose results (CTDIvol) for six centres appeared to be less for the WB and also less than the proposed NDRL for HB and the WB oncology examinations. Two of the centres had accommodated a state of art digital PET-CT which have elevated technology other than the rest of PET-CT respectively, and one centre had accommodated a Philips PET/CT of an older model, setting a low mA for the purpose of attenuation correction primarily, and with no use of AEC or ASiR. The NDRL for the second phase study (2019) showed 9.1% improvement over the 2018 NDRL result.^1^

## Discussion

This study has been able to generate data from a truly representative cross-section of Kuwait PET/CT oncology data. All the participating centres, except one, used AEC and ASiR in acquiring CT. Minimum and maximum range of mA for setting AEC were very much variable (14–209 mA & 81–400 mA respectively) for both HB and WB oncology PET-CT examinations. One centre who did not use AER, had set the mA low, in the range of 50–83 mA, with the justification that the CT scan was used for attenuation correction at the foremost. There was not much variation in the CT tube voltage setting (*i.e.* 120kV) across the eight centres.

The “mean” and “median” SL values (cm) for the HB scan were 106, 106 and for the WB were 168, 168 which appeared to be higher than the UK HB (95, 94), the Swiss HB (94, 101) and the Swiss WB (119, 128). The Swiss had only six entries, whereas KW had 200 entries for WB scans. The average male and female lengths for UK and Swiss nationals were unknown. In all cases, the CT data were used for AC and localisation, but acquisition parameters and patient doses for the eight PET-CT centre varied with a maximum of two and half fold variation in the DLP between centres. The third quartile of CTDIvol and DLP values were used to propose the local DRL (LDRL) and the first quartile of CTDIvol and DLP values were calculated to suggest the achievable LDRL for each participating centre accordingly.

**Figure 7.**
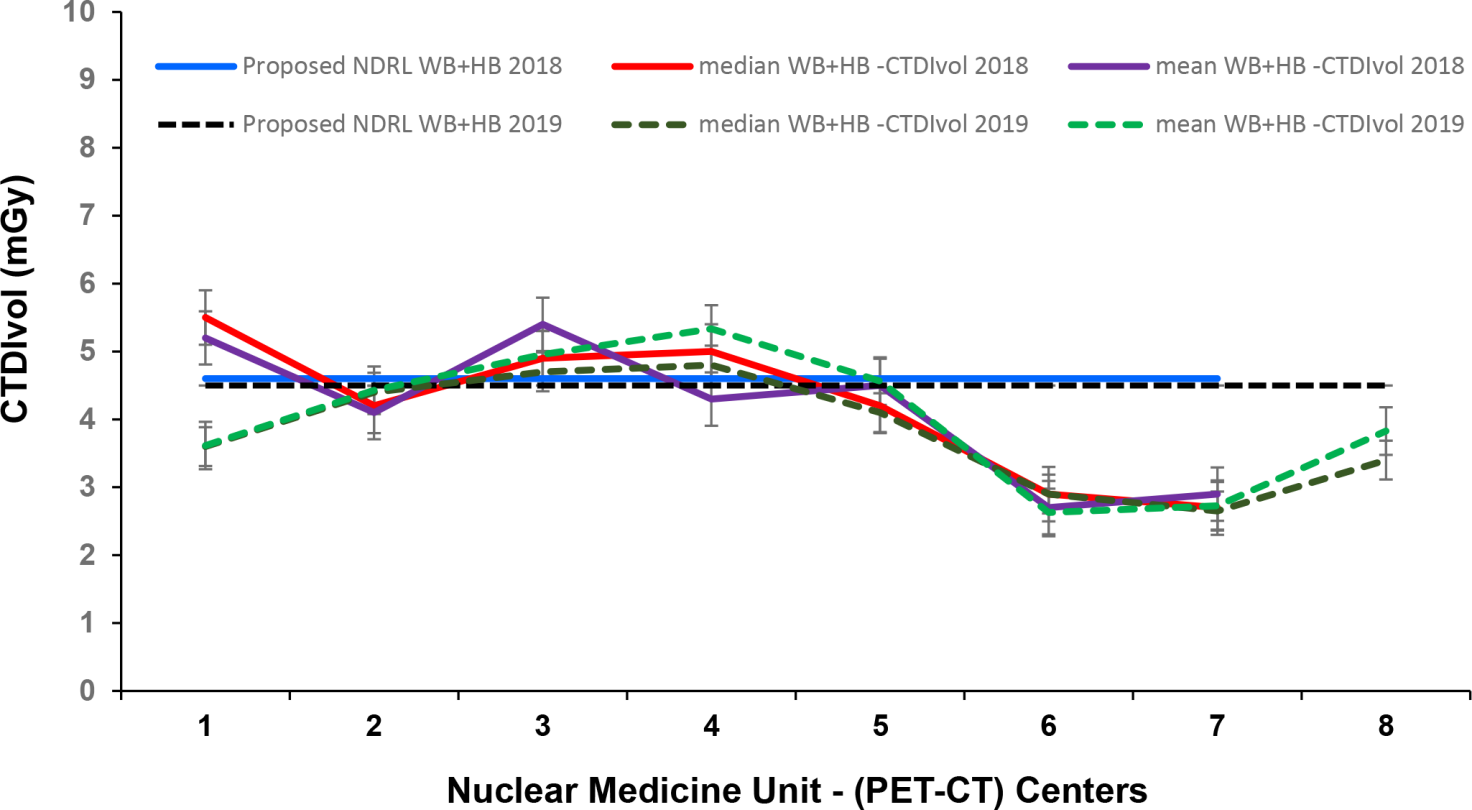
Comparison of CTDIvol (75th percentile) for WB + HB data and proposed NDRL at each PET-CT unit in 2018 and 2019. CTDI_vol_, volumetric CT dose index; HB, half body; PET, positron emission tomography; NDRL, national diagnostic reference level; WB, whole body.

**Figure 8.**
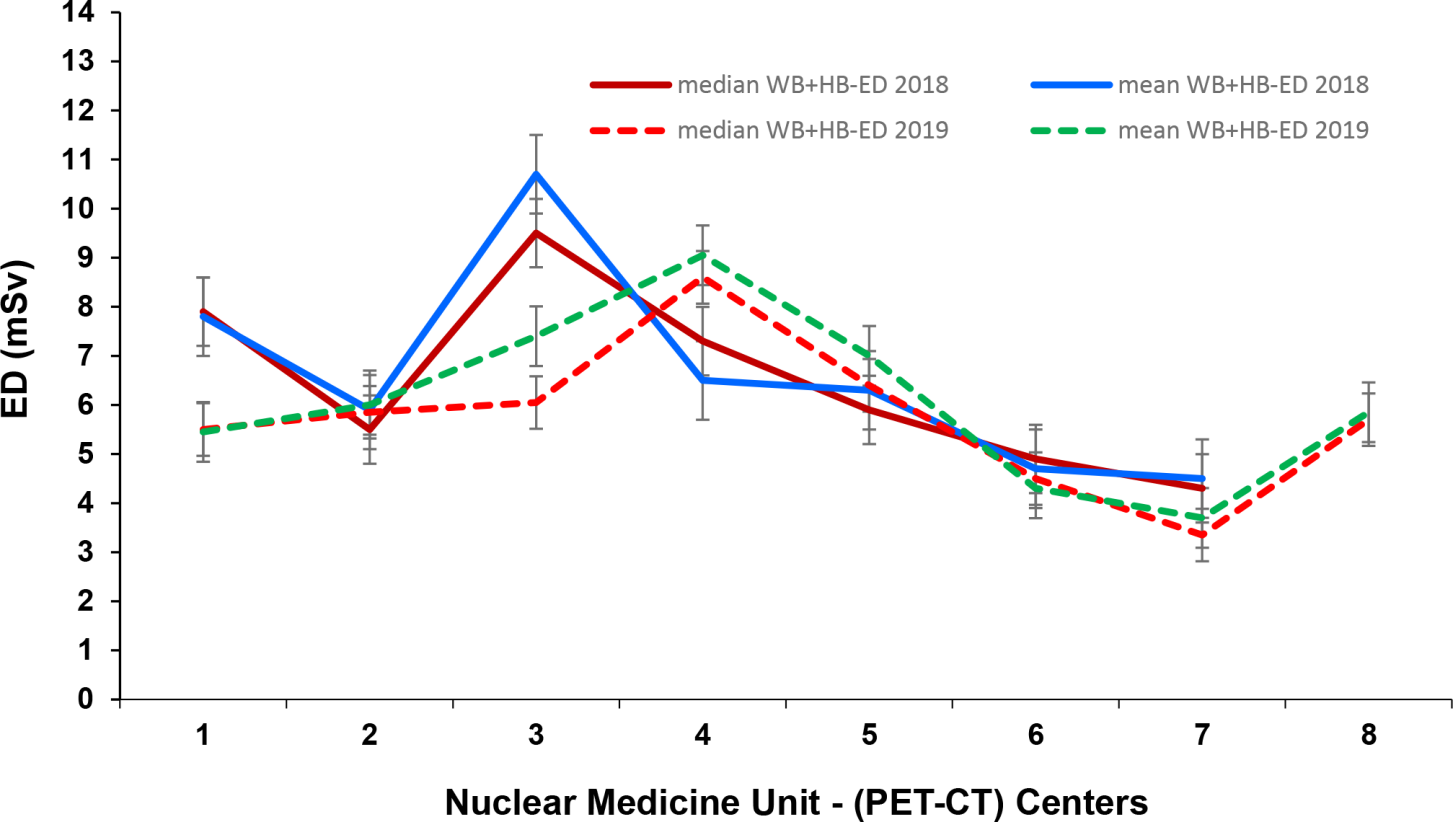
Comparison of CT ED for WB + HB data at each PET-CT unit in 2018 and 2019. ED, effective dose; HB, half body; PET, positron emission tomography; WB, whole body

Seven out of eight centres had accommodated Discovery^TM^ GE PET-CT with Optima^TM^, 64 slices CT part, including two digital GE PET-CT. The remaining scanner had a Fillips Gemini PET-CT, and they were reported using AEC. The ratio of maximum to minimum mean doses for HB and WB scans between different centres for the same clinical studies varied between 2.3–7.3 for HB and 2.1–7.3 for WB. Figures 7 and 8 presented comparative trends of CTDIvol and ED variations over 2018 and 2019.

It has been shown that for the second consecutive year (2019), there is a variation for the proposed and achievable local DRL in practice between eight centres, highlighting the need for continued assessment of national DRL and to monitor the trend of the variation with an intention to minimise the radiation dose and its impact on Kuwaiti patients.

It is expected with the final establishment of NDRLs and achievable dose, it will be possible to optimise the practice across the Kuwait. In fact, in the second phase (2019), NDRL improved by 9.1% over the 2018 NDRL and it revealed unnoticed variations in dose between the health-care facilities. The study facilitates to establish a platform for standardisation of CT dose of hybrid imaging for oncology examinations to improve the patient protection and quality care in state of Kuwait.

The published NDRLs values from other countries which may have utilised different imaging practices and technology could not be relevant to Kuwait. This study outcome will further pave the way for setting a NDRL CT part of the PET-CT examination for Kuwait populations, which will facilitate, assist and encourage to create a data bank (*i.e.* National Archive) for the future years to sever as a monitoring tool to elevate quality care for KW populations. Many advanced countries have carried out and have set up the practice for their own nations, *e.g.* UK, Swiss, France, as part of their strategy to move toward the provision of quality personal medicine including radiation dose minimisation. The authors have sighted no such data available in the Golf region and it will be beneficial to the KW Medical Imaging Community to have a reference level based on their current facilities and practice.

## Conclusions

It is anticipated that with the establishment of the NDRLs, and the continuous monitoring, it will be possible to optimise the related practice across the Kuwait and reduce this variation in the next future surveys and promote improvements in the patient protection and quality care. The audit result has further paved the way for setting a NDRL CT part of the PET-CT examination for Kuwait populations which is based on the current facilities and practice that is more realistic than using the external references, in pursue of a National Archive for the to elevate quality care for KW populations.
